# Implementation of a free water protocol at a long term acute care hospital

**DOI:** 10.1038/s41598-023-29448-5

**Published:** 2023-02-23

**Authors:** Stefanie Gaidos, Henry C. Hrdlicka, John Corbett

**Affiliations:** 1 Department of Inpatient Speech Language Pathology, Gaylord Specialty Healthcare, Wallingford, CT 06492 USA; 2 Milne Institute for Healthcare Innovation, Gaylord Specialty Healthcare, Wallingford, CT 06492 USA

**Keywords:** Rehabilitation, Health care, Quality of life

## Abstract

This feasibility study aimed to trial a Free Water Protocol (FWP) for patients with thin liquid dysphagia in the Long-Term Acute Care Hospital (LTACH) setting. Patients with dysphagia are often prescribed thickened liquids to avoid or mitigate aspiration. While this clinical intervention can minimize the risk of aspiration pneumonia (PNA), it is generally not well received by patients. As such, the goal of this study was to determine if patients who knowingly aspirate thin liquids can safely tolerate thin liquid water, and if so, to what degree of benefit. The study assessed for adverse events, fluid intake, hydration status, quality of life, and overall swallow function outcomes. These measurements were taken over a 7 day trial period using inventories, lab work, clinical judgment, and observation. Ten participants were enrolled in this study with 9 having sufficient data for analysis (n = 9). No adverse events related to the FWP were observed, and patients saw improved total fluid intake (*P* = 0.0074), swallow-related quality of life (*P* = 0.0273), and overall swallow function (*P* = 0.0002). The results in this feasibility study allowed for the hospital wide implementation of the FWP and laid out the groundwork for future studies looking at longitudinal effects of a FWP.

## Introduction

Dysphagia, the difficulty and/or inability to safely swallow, is a frequent concomitant diagnosis for adult patients in the long term acute care hospital (LTACH) setting. Dysphagia can be caused by many factors such as: neurological damage and disorders, intubation, radiation, and narrowing or damage to the esophagus^1,2^. According to the American Speech and Hearing Association, the prevalence of dysphagia spans across several acute and chronic diagnoses including: stroke, Parkinson’s disease, amyotrophic lateral sclerosis, critical illness myopathy, spinal cord injury, head and neck cancer, and other esophageal diseases^1,2^. Recent studies have also highlighted dysphagia as a finding in patients hospitalized with COVID-19, yielding greater demands for skilled, evidence-based dysphagia management^3–5^.

Dysphagia treatment typically involves both rehabilitative and compensatory techniques used to improve liquid and solid food ingestion, to preserve hydration and nutrition, and minimize the risk of a choking event or development of aspiration pneumonia (PNA)^6,7^. An exercise program may be implemented to strengthen the musculature required for more efficient bolus control and increased airway protection^6,7^. Postural techniques, such as a chin tuck strategy or head turn, may help mitigate or eliminate instances of aspiration^8^. Moreover, water, and other liquids, can be thickened to increase the viscosity to improve bolus control^9^. Although effective, these thickened liquids are not well received by patients with dysphagia, resulting in patients often refusing them outright or covertly consuming thin liquids^9,10^.

Interestingly, despite aspiration being demonstrated by videofluoroscopy, patients who consume thin liquids often do not develop aspiration PNA^11–17^. In response to this, Speech Language Pathologists (SLPs) at the Frazier Rehabilitation Institute (Louisville, KY) developed the Frazier Free Water Protocol (FWP)^18,19^. This protocol allowed patients with dysphagia to freely consume thin liquid water with supervision. Since its development, it has been largely confirmed that the FWP does not result in aspiration PNA^11–17^. The FWP has also been shown to increase overall patient satisfaction, improve patient compliance to thickened liquid diets, and improve patient hydration status due to an overall increase in liquid consumption^11,12,16,18–20^. Pulmonary complications observed in one FWP study comparing the consumption of thickened liquids and thickened liquids plus water, were determined to be the result of pre-existing neurological diagnoses, including Alzheimer's Disease, Parkinson's Disease, and congenital intellectual disability^20^.

This general absence of aspiration PNA could be due, in part, to the practice of good oral hygiene. It is widely understood that aspiration PNA is caused not by the aspiration of liquids themselves, but rather the oral bacteria that are introduced with liquids and other food particulates^21–23^. This could also be due, in part, to aquaporins. Aquaporins are water channel proteins within the lungs, which are thought to use osmosis to move aspirated water from the airways to the vascular system^24^.

We sought to introduce the FWP to our institution through a three phase feasibility campaign consisting of a staff education and preparation phase, an implementation phase, and a conclusion and reporting phase. To determine if the FWP would benefit the patient population of our LTACH, we tracked several participant characteristics including: fluid intake, hydration status, blood-urea-nitrogen/creatinine (BUN/Cr) ratio, and diet progression before and after FWP implementation. Additionally, the Functional Oral Intake Scale (FOIS) and the Swallowing Quality of Life (SWAL-QOL) assessments were administered to measure participants’ swallowing ability and perception of swallowing in response to the FWP. To distinguish between acute changes directly related to the FWP and changes expected over time due to the standard of care, we collected these measures over a 7 day period.

The goal of this report is to detail the outcomes of our feasibility trial of the FWP in the LTACH setting. We aimed to determine if patients at an LTACH level of care could safely drink thin liquids despite a known dysphagia, and if so, to what degree of benefit. Further, we outline the implementation strategy used to educate and provide staff with the necessary scientific and clinical evidence that the potential risk of aspiration PNA is outweighed by the benefit to patient rehabilitation and quality of life.

## Methods and materials

### Participants

Inpatients who exhibited a degree of pharyngeal dysphagia characterized by aspiration of thin liquids as observed by instrumental swallow studies (Modified Barium Swallow Study or Fiberoptic Endoscopic Evaluation of Swallowing) were referred to the study by their primary treating SLP. Patients referred were either on a NPO (nil-per-os; i.e. patients are not allowed to consume food or drinking orally) diet with alternate means of nutrition, or on a PO (per-os; i.e. patients are allowed food or drinking orally) diet with drink and food texture modifications as aligned with the National Dysphagia Diet (NDD)^25^.

After referral, the principal investigator used the study criteria to further determine candidacy (Table 1). To be considered, patients needed to be 18 years of age or older, capable of ambulation with a physical or occupational therapist, have overall medical stability, and show no overt signs of discomfort (i.e. excessive coughing or gagging) when drinking thin liquids. The attending physician for each patient was consulted to determine medical stability, which was primarily constituted by the patient being afebrile, hemodynamically stable, having a stable respiratory status, and no known active infection. Excluded from the study were patients with a compromised pulmonary system, including both tracheostomy and ventilator needs, patients with poor oral hygiene status, patients with an absent pharyngeal swallow reflex, and patients with a Montreal Cognitive Assessment (MoCA) score less than 17; scores less than 17 indicate the presence of moderate to severe cognitive impairment^26^. Conveniently, the Occupational Therapy department was already collecting MoCA scores for all patients at admission. We used these scores to ensure the cognitive capacity of candidates to adequately understand the rules of the protocol and give informed consent. If a patient was unable to complete the MoCA due to communication deficits, cognitive status was determined by consultation with the patient’s primary therapists, family, and analysis of etiology for hospitalization.

**Table 1:** Enrollment criteria.

Inclusion criteria:
Aspirates thin liquids as evidenced via videofleuroscopy or fiberoptic endoscopy assessment methods
18 years of age or older
Capable of ambulating with physical or occupational therapy
Medically stable including: afebrile, hemodynamically stable, stable respiratory status, no active infections nor elevated white blood counts
Able to feed self or direct feeder
Without overt signs of discomfort (i.e. excessive coughing, gagging) when drinking thin liquids
Able to sign an informed consent form

Exclusion criteria:
Compromised pulmonary system, i.e. tracheostomy and/or mechanical ventilation requirements
Absent pharyngeal swallow reflex
Medically unstable including: febrile, active pneumonia, elevated white blood counts
Poor oral hygiene status
Fluid restrictions due to cardiopulmonary issues
A montreal cognitive assessment (MoCA) score of less than 17

## Research design and setting

This was a feasibility study completed at the inpatient level of care at Gaylord Specialty Healthcare’s Gaylord Hospital in Wallingford, CT, USA. Gaylord Hospital is a 137-bed LTACH focused on medical rehabilitation across a variety of diagnoses, including: brain injury, acute and chronic pulmonary conditions, and spinal cord injuries. The average inpatient length of stay (LOS) is 24 days. As this was a feasibility study intended to investigate the implementation of this protocol at our institution and did not use an FDA regulated device or medication, this study was not registered with clinicaltrials.gov as it was determined to not meet the criteria of an applicable clinical trial^28^. Similar to prior implementation strategies^14^, this study consisted of 3 phases: a Staff Education/Preparation phase, an Implementation phase, and a Conclusion phase.

### Staff education and preparation

As our institution only had a protocol related to ice chips at this time, the importance of staff education was quickly realized as most hospital staff were either hesitant or unfamiliar with the concept of free water. During the education/preparation phase, a comprehensive interdisciplinary research committee was formed consisting of three SLPs, one occupational therapist, one physician, two registered dieticians, and one registered nurse. Committee members assisted in providing education to hospital staff in the form of in-service presentations, provision of written handouts, and establishing the protocols necessary for implementation.

Additionally, SLP committee members presented a PowerPoint Presentation at the monthly medical team meeting preceding participant recruitment. This presentation contained information related to the rationale behind using a FWP and a review of related research. The medical team then provided initial verbal consent to implement the FWP in the context of research. Specific team member duties were also assigned during this time. The physician was available to address medical questions or concerns as they arose. The occupational therapist was to monitor daily oral care interventions. The two registered dieticians tracked hydration status, including overall daily fluid intake. The hospital’s chief nursing officer was contacted to discuss the study and potential impact on nursing responsibilities (e.g., providing participants with water, recording daily fluid intake) and was agreeable to implementation. Nursing staff were requested to initial the oral care form each time oral care was completed and to record fluid intake for all drinks consumed by the participant each day during the tracking periods. A new order for “Free Water Protocol” was requested of the clinical informatics manager and placed in the electronic medical record system. This order contained the written rules of the protocol within it.

Concurrently, contact was made with clinicians and researchers in similar hospital settings to obtain permission to use or recreate applicable handouts for this study. Specifically, a brochure outlining a brief history of the free water protocol in addition to an oral care plan were adapted from research previously published by the G.F. Strong Rehab Centre^14^, an inpatient rehabilitation setting similarly structured to our LTACH setting. The brochure, a simple fluid tracking form, and the oral care plan were assembled in two-pocket folders. These were reviewed with staff and left in participants’ rooms upon the study start.

### Study protocol

**Figure 1:**
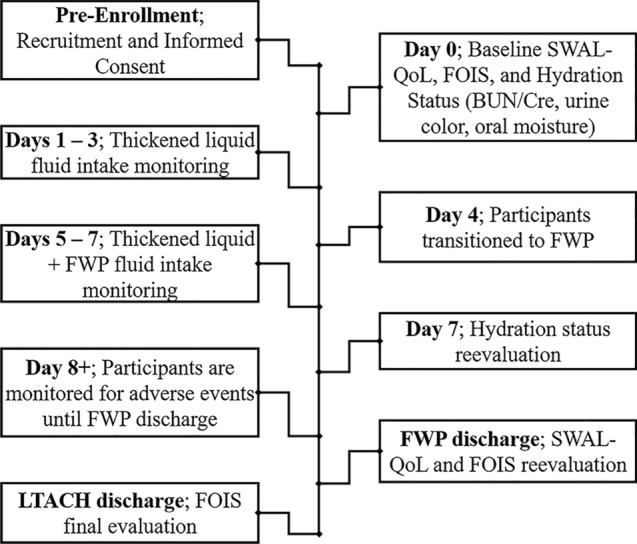
Treatment timeline. During Pre-Enrollment, inpatients identified with pharyngeal dysphagia were referred to study staff. Once recruited, baseline SWAL-QOL, FOIS, and hydration status (BUN/Cr, oral dryness, and urine color) were collected the day prior to starting the study (Day 0). During the baseline period (Days 1–3), participant fluid intake of orally delivered thickened liquids and any enteral delivered liquids were recorded. After the baseline period, participants were initiated on the FWP (Day 4). During the FWP period (Days 5–7), fluid intake of oral thickened liquids, enteral liquids, and oral thin liquid water were recorded. At the end of Day 7, participant hydration status was reevaluated. Following Day 7, intake volume was no longer recorded, but participants continued the FWP until they transitioned to a full thin liquid diet or was discontinued due to a medical contraindications; SWAL-QOL and FOIS reevaluations were collected at this time. Adverse event monitoring continued until the participant was discharged from the FWP. Upon LTACH discharge a final FOIS evaluation was collected. FOIS—functional oral-intake scale; FWP—free water protocol; LTACH—long term acute care hospital; SWAL-QOL—swallowing related quality of life.

The Implementation phase of the study used a modified Free Water Protocol based on previously published protocols^11,14,15,18,20,29^. To ensure a complete dataset could be collected given the potential for a shortened LOS for some patients, we implemented a 7 day study scheme consisting of two distinct periods: a baseline period and a FWP period (Fig. 1). During the baseline period, days 1 to 3, baseline data related to hydration status and fluid intake of any thickened liquid bolus placed in the patient’s mouth or liquids delivered via alternate means (i.e. PEG tube) were collected daily.

Day 4 was a transition day used to introduce the FWP. At this time, the attending physician was requested to put in the FWP order in the electronic medical record. Participants were given a green arm bracelet reading “Free Water Protocol” as a visual cue for staff to be aware of free water diet orders.

During the FWP period, data related to daily fluid intake and hydration were collected days 5 to 7. Once initiated, participants could have thin-liquid water in between meals (at least 30 min after meals), and in the absence of any other food or beverage. Any other food or snacks outside of meals required a minimum of a mouth rinse with water or the use of low or alcohol-free mouthwash before free water could be again consumed. Additionally, participants were provided with a new, fully filled, 32 oz plastic water container. To accurately track the amount of free water intake, participants were only permitted to drink water poured from this measured container. Prescribed PEG feedings and thickened liquids were also provided according to normal routines (e.g., on meal trays) or upon patient request; these were also recorded and included in the volume totals.

During the study and throughout the participants’ stay, a strict oral care regimen was followed. This consisted of teeth brushing and a Chlorhexidine rinse at least three times a day as aligned with the Gaylord “Oral Care Protocol” for high-pneumonia**-**risk patients. Signs related to oral care and free water rules were reviewed and hung above the participants’ beds to reinforce education and compliance across staff, families, and participants.

After day 7, participants remained on the FWP until they were: discharged from the facility; advanced to thin liquids by their primary SLP; or were required to discontinue free water intake due to medical instability. In order to monitor for adverse events, we continued to track participants until the FWP was discontinued.

### Outcome measures and data collection

The study focused on several independent and dependent variables for analysis. Independent variables collected included: participant age, diagnosis, and sex. Dependent variables collected included: incidence of adverse reaction seemingly related to the FWP; hydration status; quality of life; and functional swallow ability. To track incidence of adverse reaction, a participant’s medical chart and clinical presentation were monitored daily. This included review of documentation for abnormal lung sounds, white blood cell count, temperatures, and chest X-ray results if requested by the treating physician. Aggregately, this indicated aspiration PNA and would be recorded as such in the physician’s daily note^30,31^.

Hydration status was tracked by the registered dieticians using several measures over the course of the seven day study period. These consisted of pre- and post-ratings of oral hygiene status (moist, dry, or cracked), urine color (dark or yellow/clear), and laboratory findings for BUN/Cr. For BNU/Cr, measurement within the range of 6–22 mg/dL were considered normal^32^. An overall impression of “dehydrated” was given if 2 out of 3 measures were deviant from the norm as per these internally created clinical criteria and guidelines from our registered dietician team. Fluid intake was also tracked to assess for any substantial changes when on the protocol.

To assess for quality of life changes, we requested each participant complete the SWAL-QOL scale upon study enrollment, and again when the study ended^33^. The SWAL-QOL is a 44-item questionnaire designed to assess a patient’s perceived satisfaction and challenges in regard to eating using a five point ordinal scale. The lower the score sum, the more impactful or difficult swallowing was perceived. If a participant exhibited visual, reading, or writing deficits that may impact authenticity of responses, the form was read aloud to the participant and responses recorded by the investigator.

The final dependent variable in this study was functional eating ability. We used the FOIS , a psychometric tool that assigns numeric values based on an individual’s means and type of nutritional intake^34^. Clinicians gave participants a baseline FOIS score upon study enrollment, a second score upon FWP discontinuation, and a third at LTACH discharge. The FOIS was already being used by the inpatient Speech Therapy department and was available for electronic documentation.

### Statistical analysis

As this was a feasibility study, a power analysis and sample size estimation were not conducted. Data was analyzed using GraphPad Prism version 9.1.1 (GraphPad Software, San Diego, CA). Shapiro–Wilk Test for normality was first used. The only normally distributed collected dataset was the total fluid intake volume. Total fluid intake measurements were averaged for each period and subsequently compared; paired two-tailed Student’s *t*-test was used to determine if differences existed between baseline and FWP periods. The rest of the datasets were abnormally distributed and evaluated using nonparametric tests. BUN/Cr ratio and SWAL-QOL results were analyzed using a Wilcoxon matched-pairs signed rank test to determine if differences existed between baseline and FWP periods. The three measurements collected for FOIS were compared using Kruskal–Wallis analysis of variance (ANOVA) and Dunn’s multiple comparisons test.

### Ethical standards

Before participant recruitment, the study was reviewed and approved by the Gaylord Hospital Institutional Review Board (Wallingford, CT, USA) to ensure the study complied with the ethical-standards set by the Declaration of Helsinki. Written informed consent was delivered to and collected from each participant before study activities were conducted.

## Results

### Participant characteristics and nutritional status

The study was comprised of participants with a wide range of admitting diagnoses as listed in Table 2. Participants were recruited between August 2018 and February 2020. Consistent with an inpatient rehabilitation program, all participants were transferred to the LTACH from an acute care hospital. After providing informed consent, ten participants were then enrolled in the study. Due to the strict inclusion and exclusion criteria, the ten participants that consented to this study were the only patients that were approached. One participant who was originally enrolled, was excluded from data analysis. Due to unrelated medical issues that ultimately required a transfer from our facility, we were uncertain of the validity and quality of the data collected from this individual. All data are representative of the other nine individuals.

**Table 2:** Participant characteristics.

Subject	Age (y)	LTACH LOS (d)^a^	FWP duration (d)^b,c^	Sex	Diagnosis	Diet		
						Study enrollment	FWP discontinued	LTACH Discharge
1	50	32	10	M	MT^d^	NDD2/NTL^e,f^	Regular	Regular
2	65	45	18	M	SCI^g^	NDD2/NTL with PEG supplement^h^	NDD3/Thins	Regular
3	67	18	9	M	ARF^i^	NDD2/NTL	Regular	Regular
4	41	25	10	M	MT	NDD3/NTL	Regular	Regular
5	65	23	17	M	Stroke	NDD1/NTL	NDD3/Thins	Regular
6	33	50	11	F	Stroke	NDD2/NTL	NDD2/NTL	NDD2/NTL
7	66	24	8	M	Stroke	NPO s/p PEG^j,k^	NDD3/Thins	Regular
8	73	75	13	F	ARF	NDD2/NTL	Regular	Regular
9	19	18	9	M	ARF	NDD1/NTL	Regular	Regular
Mean (95% CI)	53 (39, 68)	34 (20,49)	12 (9, 14)					

^a^*LTACH* long-term acute care hospital; ^c^*FWP* free water protocol; ^d^*MT* multiple trauma; ^e^*NDD* national dysphagia diet; ^f^*NTL* nectar thick liquids; ^g^*SCI* spinal cord injury; ^h^*PEG* percutaneous endoscopic gastrotomy tube; ^i^*ARF* acute respiratory failure; ^j^*NPO* nil per os; ^k^*s/p* status post.

^b^Participants were followed beyond the seven day study period to monitor for the incidence of any adverse events related to the FWP. Monitoring was discontinued once the FWP was discontinued due to a transition to a full thin liquid diet or a medical contraindications, or the participant was discharged from the LTACH setting.

Consisting of 2 females and 7 males, the mean (95% CI Lower Limit, 95% CI Upper Limit) age of the 9 participants included in analysis was 53 (39, 68) years. The admitting diagnoses for these participants included: stroke (3 cases), acute respiratory failure (3 cases), multi-trauma (2 cases), and spinal cord injury (1 case). The mean (95% CI LL, UL) participant LTACH LOS was 34 (20, 49) days, and the mean (95% CI LL, UL) length of FWP participation was 12 (9, 14) days (Table 2). At the start of the study, 1/9 participants entered the study as NPO with alternate means of nutrition by PEG tube, 1/9 entered the study with a NDD diet with nectar-thick-liquids (NTL) and PEG tube supplement, and the 7/9 remaining participants were prescribed a NDD/NTL diet. All but one participant exited the study with advanced diet levels, with 5/9 classified as “Regular Consistency”; all but one participant was discharged from the LTACH with a Regular Consistency diet (Table 2). Participants were tracked beyond the 7 day study period to monitor for adverse events; no adverse events related to the FWP were observed during this time. They all received daily occupational, physical, and speech therapy 5 to 6 days a week as appropriate. Per standard procedures, either a physiatrist or a hospitalist was assigned to the patient for daily rounds and medical monitoring.

### Evaluation of participant fluid intake and hydration status following FWP implementation

**Figure 2:**
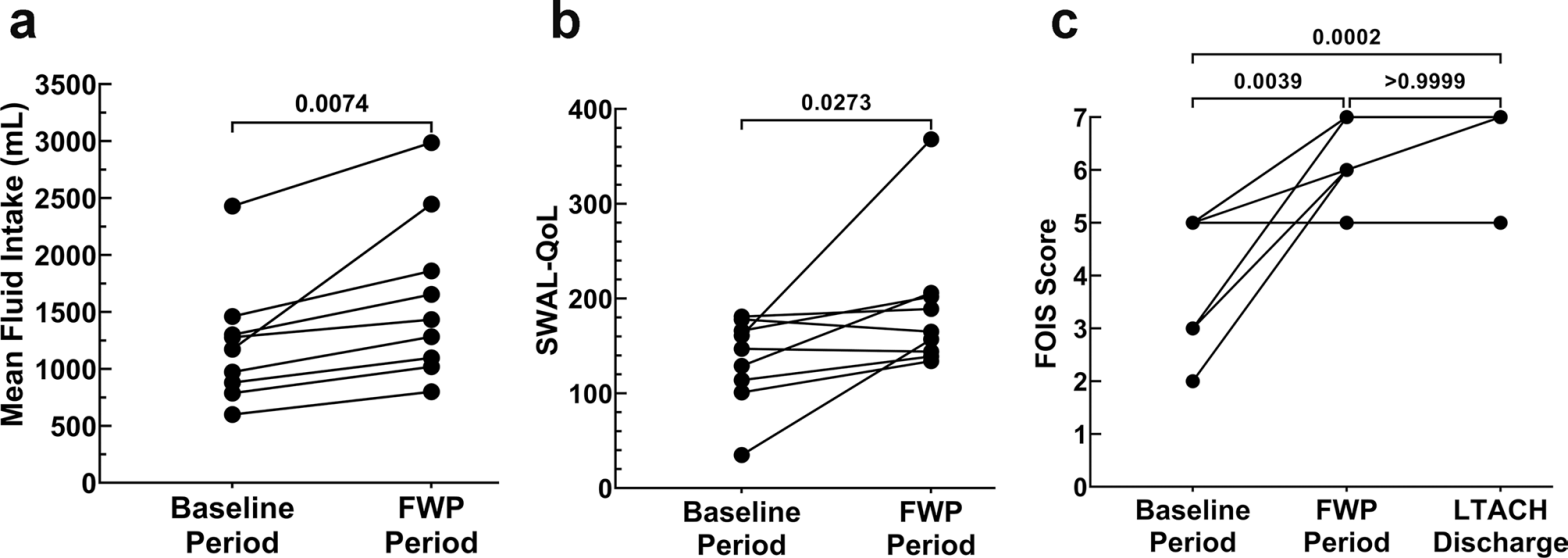
Participant outcomes. (**A**) The mean fluid intake was recorded for the Baseline period, days 1–3, and for the FWP period, days 5–7; mean fluid intake for each period is shown. Baseline fluid intake consisted of orally delivered thickened liquids and any enteral delivered liquids. FWP consisted of oral thickened liquids, enteral liquids, and oral thin liquid water. (**B** and **C**) Participant quality of life related to swallowing (**B**) and swallowing function via FOIS (**C**) were assessed just prior to the start of the Baseline period and again when participant’s discharged from the FWP; FOIS was recorded a final time upon LTACH discharge. Before and after plots were used to visualize the progression of scores between time points; n = 9. FOIS—functional oral-intake scale; FWP—free water protocol; LTACH—long term acute care hospital; mL—milliliter; SWAL-QOL—swallowing related quality of life.

Fluid intake volumes were only tracked during the baseline (days 1–3) and FWP (days 5–7) periods, and included all liquid that was delivered by tube feeding, thickened liquids, and, during the FWP, any thin liquid water participants chose to consume. With a mean (95% CI LL, UL) increase of 411 (145, 677) milliliters (mL), fluid intake volume significantly increased from a baseline volume of 1209 (799, 1619) mL during days 1 to 3, to a FWP volume of 1620 (1072, 2168) mL (*P* = 0.0074) during days 5 to 7 (Table 3 and Fig. 2a).

**Table 3:** Average fluid intake volume.

Participant	Baseline period, average volume (mL)^a^	FWP period, average volume (mL)^b,c^	Volume change (mL)
1	973 (160)	1283 (216)	310
2	1278 (242)	1433 (233)	155
3	600 (159	800 (183)	200
4	1173 (181)	2447 (421)	1274
5	880 (302)	1097 (237)	217
6	787 (363)	1020 (159)	233
7	1300 (60)	1654 (104)	354
8	2430 (286)	2987 (390)	557
9	1460 (394)	1860 (275)	400
Mean (95% CI)^e^	1209 (799, 1619)	1620 (1072, 2168)^e^	411 (145, 677)

^a^Mean (SD) for fluid intake over baseline days 1–3; includes all orally delivered thickened liquids and enteral delivered liquids. ^b^Mean (SD) for fluid intake collected over FWP days 5–7; includes all orally delivered thickened liquids, thin liquid water, and enteral delivered liquids. ^c^*FWP* free water protocol. ^d^95% *CI* 95% confidence interval. ^e^Significantly different compared to baseline; *P* = 0.0074.

Participant hydration status was then measured using multiple variables, including: serum BUN/Cr ratio, urine color, and oral hydration status (Table 4). At baseline, with a mean (95% CI LL, UL) BUN/Cr ratio of 27 (15, 38) mg/dL, only 33% (3/9) of participants had BUN/Cr ratios that were considered to be within-normal limits (i.e. within a range of 6–22 mg/dL)^32^. Following the FWP period, although 33% (3/9) of participants observed slight increases in their BUN/Cr ratio, indicating a slight worsening of their hydration, 78% (7/9) presented with BUN/Cr ratios considered to be within normal limits. Despite this moderate improvement, the mean (95% CI LL, UL) BUN/Cr ratio of 21 (10, 32) mg/dL following the FWP was not significantly different (*P* = 0.1406) to baseline (Table 4). Furthermore, descriptive measures were also used to assess hydration status. At the beginning of the study period, 11% (1/9) of participants presented with a dry oral cavity and 22% (2/9) presented with dark urine. These observations, paired with elevated BUN/Cr ratios, indicated that 22% (2/9) of the participants were dehydrated at the beginning of the study period. Following the FWP period, all participants were deemed to be hydrated (Table 4).

**Table 4:** Hydration status.

Participant	Baseline hydration status^a^				Hydration status following FWP^b,c^			
	BUN/Cr (mg/dL)^d^	Urine	Oral	Impression	BUN/Cr (mg/dL)	Urine	Oral	Impression
1	36	Yellow	Moist	Hydrated	24	Yellow	Moist	Hydrated^e^
2	29	Yellow	Dry	Dehydrated	22^f^	Yellow	Moist	Hydrated
3	42	Dark	Moist	Dehydrated	19^f^	Yellow	Moist	Hydrated
4	9^f^	Dark	Moist	Hydrated	9^f^	Yellow	Moist	Hydrated^e^
5	13^f^	Yellow	Moist	Hydrated	15 f.	Yellow	Moist	Hydrated^e^
6	8^f^	Yellow	Moist	Hydrated	13^f^	Yellow	Moist	Hydrated^e^
7	49	Yellow	Moist	Hydrated	57	Yellow	Moist	Hydrated^e^
8	30	Yellow	Moist	Hydrated	16^f^	Yellow	Moist	Hydrated^e^
9	24	Yellow	Moist	Hydrated	12^f^	Yellow	Moist	Hydrated^e^
Mean (95% CI)	27 (15, 38)				21 (10, 32)^g^			

^a^Collected prior to the baseline period days 1–3; ^b^Collected following the FWP period days 5–7; ^c^*FWP* free water protocol; ^d^*BUN/Cr* blood urea nitrogen/creatinine; ^e^No change in overall hydration status; ^f^BUN/Cr falls within normal limits of 6 – 22 mg/dL; ^g^Not significantly different to baseline; mean (95% CI) difference =  − 6 (− 14, 2), *P* = 0.1406.

### Assessment of swallowing related quality of life and function following FWP implementation

To assess swallowing related quality of life, participants completed the SWAL-QOL at the start and end of the seven day study period. With 7/9 (78%) participants showing an improvement, the mean (95% CI LL, UL) SWAL-QOL scores significantly increased from 135 (99, 171) to 189 (134, 245) (*P* = 0.0273), a 55 (0.3, 109) point increase (Table 5 and Fig. 2b). Participants’ functional eating ability was then measured using the FOIS at baseline, upon FWP discontinuation, and at LTACH discharge. From baseline, the mean (95% CI LL, UL) FOIS scores of 4 (3, 5) significantly improved to 6 (6, 7) at FWP discontinuation (*P* = 0.0039). From FWP discontinuation to LTACH discharge, mean (95% CI LL, UL) FOIS scores only marginally improved to 7 (6, 7). Although FOIS scores at LTACH discharge were not different to FWP discontinuation (*p* > 0.9999), they were significantly different than the baseline (*P* = 0.0002) (Table 6 and Fig. 2c).

**Table 5:** Swallowing quality of life (SWAL-QoL).

Participant	Baseline^a^	Following FWP period^b,c^	Score Change
1	129	206	77
2	147	144	− 3
3	178	165	− 13
4	161	368	207
5	181	189	8
6	101	134	33
7	114	139	25
8	166	202	36
9	35	157	122
Mean (95% CI)	135 (99, 171)	189 (134, 245)^d^	55 (0.3, 109)^e^

^a^Collected prior to the days 1–3 baseline collection period; ^b^Collected following the days 5–7 FWP period; ^c^FWP: free water protocol; ^d^Significantly different compared to baseline using Wilcoxon matched pairs test; *P* = 0.0273; ^e^Differences in rounding may give the appearance of math errors.

**Table 6:** Functional oral intake scale (FOIS).

Participant	Baseline^a^	FWP discontinued^b,c^	LTACH discharge^d,e^
1	5	7	7
2	3	6	7
3	5	7	7
4	5	7	7
5	5	6	7
6	5	5	5
7	2	6	7
8	3	7	7
9	5	7	7
Mean (95% CI)	4 (3, 5)	6 (6, 7)^f^	7 (6, 7)^g^

^a^Collected prior to the days 1–3 baseline collection period; ^b^Collected once the FWP was discontinued due to improvements, discharge, or destabilization; ^c^*FWP* free water protocol; ^d^Collected at time of participant discharge; ^e^*LTACH* long term acute care hospital; ^f^Significantly different compared to baseline using Kruskal Wallis Test; *P* = 0.0039; ^g^Significantly different compared to baseline using Kruskal–Wallis﻿ ANOVA and Dunn's Multiple Comparison Test; *P* = 0.0002.

## Discussion

### Principle findings

In this feasibility study, we have shown that the FWP was a safe means for improving hydration, quality of life, and overall swallow function in a small sample of participants in an LTACH rehabilitation program. Significant changes included improvements to fluid intake, functional eating ability, and quality of life. Indeed, through this feasibility study we found that participants with a variety of underlying diagnoses at admission, including stroke, multi-trauma, spinal cord injury, and acute respiratory failure benefited from the FWP. This leads us to believe that many conditions which cause pharyngeal dysphagia, including those related to COVID-19, may benefit from the FWP.

Moreover, the findings of this study supports prior literature and indicates that patients who are higher functioning (i.e. medically stable, able to ambulate with the assistance of a physical or occupational therapist, and cognitively fit), and adhere to a strict oral hygiene intervention plan, are unlikely to develop aspiration PNA and will benefit from a FWP^11–17,20^. This was shown by significant improvements to swallow related quality of life scores when patients participated in our FWP, which aligns with the findings of Carlaw, et al.^14^. Whereas some prior reports demonstrated no difference in total fluid intake between the FWP and thick liquid diet^11,12,17^, our participants observed a modest, yet significant increase in total fluid intake, which is similar to other findings^14,20^. Participants in our study also showed significant improvements to functional eating ability, as well as modest, though non-significant, decreases in BUN/Cr ratios, which are indicative of improved hydration levels, which aligns with the findings of Murray, et al.^16^.

### Inclusion and exclusion criteria development

Great care and deliberation was taken when developing our inclusion/exclusion criteria. Given this was the first time a FWP was to be trialed at our institution, we did not want to put more vulnerable patients at risk while we worked through this feasibility study, and therefore we opted for a conservative approach. Specifically, we felt including an ambulation requirement was both medically essential for pulmonary clearance and in alignment with our hospital’s core mission for patient mobilization. It has also been shown that low mobility is a risk factor for developing aspiration PNA for those enrolled in a FWP^20^.

Further, we ensured that each participant’s cognitive function was in the normal to mildly impaired range according to the MoCA. The only exception to this was the inclusion of a participant whose stroke took place in the brainstem, resulting in a severe dysarthria and inability to complete the MoCA. However, after consulting with the participant’s primary therapy team, family, and taking in consideration that brainstem stroke etiologies typically do not impact cognition, it was confidently determined that the patient’s cognition was sufficient for giving informed consent and study participation.

### Omitted participant

Despite the conservative criteria, one participant was recruited to the study who was ultimately withdrawn due to the onset of acute medical concerns. Shortly after study enrollment, this participant developed a moderate to large pleural effusion which resulted in significant chest wall pain with coughing. Consequently, this participant drank minimal amounts of water during the study period in fear of cough elicitation. The multidisciplinary research committee analyzed these medical changes to determine if there was any connection with the study, and to determine if the participant should be withdrawn as overt discomfort with water consumption was a study exclusion criterion. Further analysis of the participant’s past medical history, in conjunction with discussions with the medical team, led investigators to conclude that the pleural effusion was likely unrelated to the FWP. Rather, the patient’s history of laryngeal cancer with radiation-induced vasculopathy, generalized deconditioning related to a new cerebrovascular accident, and perhaps a previous undocumented PNA could have all contributed to the onset of pulmonary issues. Additionally, the new effusion was diagnosed via a chest computed tomography angiography ten days after beginning water consumption with no suggestion of a pneumonia mentioned in the results report (atelectasis and subpleural nodules were identified as findings). This participant was ultimately transferred back to an acute care facility for more invasive intervention and was formally discharged from the study. The results of this case were then not included in data analysis. For future studies, including an exclusion criteria for considering pre-existing or recent conditions that may affect the success of the FWP should be considered.

### Study limitations

With these findings, limitations and considerations that may impact interpretation of these results need to be discussed. These include the conservative inclusion/exclusion criteria as previously discussed, the small sample size, and the brief length of the pre/post fluid intake tracking. Future studies investigating the safety, efficacy, or impact of a FWP should aim to recruit more participants. The sample size was purposely kept small and inclusion/exclusion criteria kept conservative to minimize any undue risk while the protocol was being trialed. As cited in previous literature, the FWP is appropriate for high level functioning patients (i.e. medically stable, able to ambulate with the assistance of a physical or occupational therapist, and cognitively fit) that meet a careful inclusion/exclusion criteria^12^. Our clinical population does not often reflect the target population for a FWP, as we frequently see medically complex patients with cognitive deficits and pulmonary complications. This is reflected in our low sample size over a two year period. As such, it is possible that in a full-scale study, the incidence of adverse events may be higher. Not only is the potential for adverse events higher in a larger scale study, but the conclusions we have reached are difficult to generalize to a larger population due to the small sample size of our study. Additionally, it is possible that the observed effect sizes may be greater or lesser in a larger study. Compared to other studies, we used a shorter pre/post fluid intake tracking period of seven days^11,14,15,18,29^. This was done to not only account for acute changes in participant status, but also to account for the possibility of unexpected early discharges during this trial, and the nature of a short length of stay in the LTACH setting.

It should also be noted that during the FWP period, several participants reported their water intake was limited by their demanding therapy schedules, meaning they were unable to drink water from the designated container in their rooms while in the therapy gym. Several things can be hypothesized from this observation. First, incorporating a mobile container such as a capped bottle that can accompany participants to the therapy floor might be useful for providing water throughout the day as desired. Second, by collecting more measurements overtime, implementing a longer tracking period may allow for a more accurate and reliable comparison of the FWP to the standard of care and pre-existing literature. Third, since participants subjectively were unable to drink as much during Monday through Friday and sometimes Saturday due to therapy, depending on when the FWP tracking period began, it is possible that fluid volume intake could be higher due to a lighter therapy regimen on the weekends. However, this is not certain and would need to be evaluated in a follow-up study.

### Study strengths

While the limitations of this study were recognized, there were several factors we felt were highly effective in successful implementation of this FWP. Specifically, the early education campaign to nursing staff and physicians was vital given the paradigm shift that would occur in rationalizing acceptance for potential aspiration events. We found the protocol was more readily dispersed and accepted once the SLP team members presented previous free water research and rationale. Furthermore, creating an interdisciplinary team that included representatives from every aspect of patients’ care was essential in getting initial acceptance and proper carryover across hospital departments. It also proved both beneficial and respectful to contact the chief nursing officer in advance of the study to clearly outline additional roles requested of nursing staff to maximize preparations and carryover.

### Staff re-education and dissemination of findings

Presenting post-study data analysis and conclusions to the physicians also proved valuable. The physicians expressed a good understanding of the study’s protocol and outcomes, and were in agreement for a hospital wide standard implementation with clinical judgment. Additionally, to further disseminate the findings, hospital wide in-services were given to all staff involved with patient care. The education of inpatient SLP staff was a priority as they would be primarily responsible to determine if a patient was a candidate for the FWP going forward. Prior to implementation, SLPs were encouraged to review the FWP protocol with their patients’ nurses, give patients a green FWP arm bracelet to make other staff aware, and give patients copies of the educational materials used in the study. Investigators were available to field any questions related to the protocol after the study was completed.

### Future directions

Throughout this study, we have identified two potential follow-up studies that we believe would be valuable. First, the FOIS scale^35^ data collected suggests that there is a chance the FWP may directly improve functional eating ability by allowing patients with dysphagia to “practice” drinking water before being challenged with a thin liquid diet. While not confirmed by the data collected from this study, we believe it would be beneficial to explore the effects of a FWP on functional eating ability. The rationale for this is based on the belief that improving general swallow function would improve the swallow musculature needed to safely consume regular thin liquids and solid food. Second, staff members working with participants subjectively reported observable changes in participants’ affect and mood once able to drink water. Several participants also reported that they believed their dysphagia would not have resolved as quickly if it was not for being able to drink water. While we did not evaluate the effect of FWP on affect, mood, and the recovery rate, we believe this anecdotal report may deserve further attention in a follow-up study to confirm or deny these subjective reports.

## Conclusion

In this study, our primary goal was to determine the feasibility of implementing a FWP in the LTACH setting. Our results show that, with the inclusion of a diverse interdisciplinary team, hospital wide acceptance and implementation of the FWP was achievable. Maybe more importantly, the FWP was also found to be safe with no incidence of aspiration PNA being reported within the study population. Within this population we also observed significant increases in quality of life, total fluid intake, and overall swallow function. However, these results should be interpreted cautiously due to the small sample size and other limitations outlined above. In the end this feasibility study was successful and led to the adoption of the FWP in our LTACH setting, and it may provide the evidence and protocol needed for other LTACH settings to do the same. Furthermore, given the on-going COVID-19 pandemic, it is our opinion that the FWP should be considered, and may be useful, for patients undergoing rehabilitation for COVID-19 related dysphagia. Going forward, this study laid the groundwork for future studies regarding longitudinal effects of the FWP and a more detailed investigation regarding swallow function following the FWP in the LTACH setting.

## Data Availability

Copies of the detailed treatment protocol is available upon request. Requests for copies of the de-identified datasets will be considered upon request as appropriate. To make a request, please contact Henry C. Hrdlicka (hhrdlicka@gaylord.org).

## Abbreviations

ARF: Acute respiratory failure
BUN/Cr: Blood urea nitrogen/creatinine
CI: Confidence interval
FOIS: Functional oral intake scale
FWP: Free water protocol
HTL: Honey thick liquids
LTACH: Long-term acute care hospital
MoCA: Montreal cognitive assessment
MT: Multi-trauma
NDD: National dysphagia diet
NPO: Nil-per-os
NTL: Nectar thick liquids
PNA: Pneumonia
SCI: Spinal cord injury
SLP: Speech language pathologist
SWAL-QoL: Swallowing-quality of life
